# A pro-diabetogenic mtDNA polymorphism in the mitochondrial-derived peptide, MOTS-c

**DOI:** 10.18632/aging.202529

**Published:** 2021-01-19

**Authors:** Hirofumi Zempo, Su-Jeong Kim, Noriyuki Fuku, Yuichiro Nishida, Yasuki Higaki, Junxiang Wan, Kelvin Yen, Brendan Miller, Roberto Vicinanza, Eri Miyamoto-Mikami, Hiroshi Kumagai, Hisashi Naito, Jialin Xiao, Hemal H. Mehta, Changhan Lee, Megumi Hara, Yesha M. Patel, Veronica W. Setiawan, Timothy M. Moore, Andrea L. Hevener, Yoichi Sutoh, Atsushi Shimizu, Kaname Kojima, Kengo Kinoshita, Yasumichi Arai, Nobuyoshi Hirose, Seiji Maeda, Keitaro Tanaka, Pinchas Cohen

**Affiliations:** 1 Graduate School of Health and Sports Science, Juntendo University, Chiba, Japan; 2 Leonard Davis School of Gerontology, University of Southern California, Los Angeles, CA 90089, USA; 3 Department of Administrative Nutrition, Faculty of Health and Nutrition, Tokyo Seiei College, Tokyo, Japan; 4 Department of Preventive Medicine, Faculty of Medicine, Saga University, Saga, Japan; 5 Faculty of Sports and Health Science, Fukuoka University, Fukuoka, Japan; 6 Japan Society for the Promotion of Science, Tokyo, Japan; 7 Department of Preventive Medicine, Keck School of Medicine of University of Southern California, Los Angeles, CA 90033, USA; 8 Division of Endocrinology, Diabetes and Hypertension, Department of Medicine and the Iris Cantor-UCLA Women's Health Research Center at the David Geffen School of Medicine, University of California, Los Angeles, CA 90095, USA; 9 Division of Biomedical Information Analysis, Iwate Tohoku Medical Megabank Organization, Disaster Reconstruction Center, Iwate Medical University, Iwate, Japan; 10 Department of Integrative Genomics, Tohoku Medical Megabank Organization, Tohoku University, Miyagi, Japan; 11 Center for Supercentenarian Medical Research, Keio University School of Medicine, Tokyo, Japan; 12 Faculty of Health and Sport Sciences, University of Tsukuba, Tsukuba, Ibaraki, Japan

**Keywords:** diabetes, mitochondrial DNA, polymorphism, MOTS-c, insulin resistance

## Abstract

Type 2 Diabetes (T2D) is an emerging public health problem in Asia. Although ethnic specific mtDNA polymorphisms have been shown to contribute to T2D risk, the functional effects of the mtDNA polymorphisms and the therapeutic potential of mitochondrial-derived peptides at the mtDNA polymorphisms are underexplored. Here, we showed an Asian-specific mitochondrial DNA variation m.1382A>C (rs111033358) leads to a K14Q amino acid replacement in MOTS-c, an insulin sensitizing mitochondrial-derived peptide. Meta-analysis of three cohorts (n = 27,527, J-MICC, MEC, and TMM) show that males but not females with the C-allele exhibit a higher prevalence of T2D. In J-MICC, only males with the C-allele in the lowest tertile of physical activity increased their prevalence of T2D, demonstrating a kinesio-genomic interaction. High-fat fed, male mice injected with MOTS-c showed reduced weight and improved glucose tolerance, but not K14Q-MOTS-c treated mice. Like the human data, female mice were unaffected. Mechanistically, K14Q-MOTS-c leads to diminished insulin-sensitization *in vitro*. Thus, the m.1382A>C polymorphism is associated with susceptibility to T2D in men, possibly interacting with exercise, and contributing to the risk of T2D in sedentary males by reducing the activity of MOTS-c.

## INTRODUCTION

The prevalence of type 2 diabetes mellitus (T2D) is growing dramatically. Over 400 million individuals were diagnosed with T2D worldwide in 2015, and the International Diabetes Federation projects that at least 600 million people will need to be treated for T2D by 2040 [1]. However, treating T2D is challenging because the disease etiology is genetically heterogeneous and varies among ethnicities [2]. For example, a recent meta-analysis showed that T2D is 72% heritable (95% CI: 61-78%) [2], and the Western Pacific Region – including China and Japan – makes up ~37% of the total T2D diagnoses in the world [1]. This is particularly noteworthy because they have a higher susceptibility to T2D compared to Caucasians although East Asian populations have lower mean body mass index (BMI) than Caucasian populations [3]. However, when matched for BMI, East Asians have a greater percentage of body fat and a tendency for increased visceral adiposity [4]. T2D in Asian patients is characterized by early beta cell dysfunction, in contrast to Caucasian populations that have more insulin resistance [5]. Thus, ethnicity-based DNA variations likely influence the pathogenesis of T2D.

While diabetes syndromes directly caused by mutations in mtDNA are extremely rare [6], several genetic analyses reveal that mtDNA polymorphisms contribute to T2D risk in both European and Asian populations [7, 8]. Notably, mtDNA sequences are more varied by ethnicity compared to nuclear DNA sequences, due to the 10-times higher mutation rate of mtDNA compared to nuclear DNA [9]. The human mitochondrial genome consists of 16,569-base pairs and encodes 37 genes including 13 full size proteins involved in oxidative phosphorylation, 2 rRNA, and 22 tRNA genes that are necessary for protein synthesis within the mitochondria. Genetic polymorphisms in the mtDNA could potentially affect cell metabolism [10] leading to alterations in insulin signaling or beta cell function and may explain why T2D prevalence is different between ethnicities. As a result, certain mtDNA polymorphisms could contribute to the T2D prevalence in different ethnicities. Although the association is strongly studied in population genetics, the functional effects of the mtDNA polymorphisms are underexplored. Sequence variation in mtDNA may cause slight differences in the function of the respiratory chain, free radical production, the alteration of mitochondrial matrix pH, and intracellular calcium levels [11].

Mitochondrial-derived peptides (MDPs) are biologically active peptides derived from small open reading frames (ORF) in the mitochondrial genome [12–16]. Emerging studies suggest that mtDNA polymorphisms could impact mitochondrial-derived peptides levels. For example, the m.2706A>G variant in the ORF encoding the mitochondrial-derive peptide humanin is associated with accelerated cognitive aging and a decrease in circulating humanin levels [17].

Another mitochondrial-derived peptide encoded from the 12S rRNA in the mtDNA is called mitochondrial open-reading-frame of the twelve S rRNA – type c (MOTS-c) [14]. MOTS-c is a 16-amino acid peptide expressed in multiple tissues, including skeletal muscles, and is detected in plasma. MOTS-c activates AMPK and acts as an insulin sensitizer [14]. MOTS-c regulates insulin sensitivity and metabolic homeostasis in the skeletal muscle of mice [14]. MOTS-c reduces weight gain and decreases fat accumulation in the liver in high-fat diet-induced obese mice. MOTS-c levels correlate with muscle fiber composition in men [18], leading some to calling it an “exercise-mimetic peptide” [19]. MOTS-c also prevents ovariectomy-induced metabolic dysfunction [20]. MOTS-c levels are correlated with insulin resistance, and circulating MOTS-c levels are reduced in obese male (but not female) children [20, 21]. MOTS-c levels are inversely correlated with markers of insulin resistance and obesity including BMI, waist circumference, waist-to-hip ratio, fasting insulin level, HOMA-IR, HbA_1c_ [21].

The East Asian-specific mtDNA Single Nucleotide Polymorphism (SNP), m.1382A>C (rs111033358), causes an amino acid replacement from Lys (K) to Gln (Q) at the 14th amino-acid residue in the MOTS-c peptide [22]. This SNP is mainly found in haplogroup D4b2. A previous study showed that the mitochondrial haplogroup D4b, which includes D4b2, is associated with an increased risk of T2D (*p* = 0.002) with an odds ratio of 3.55 (95% confidence interval 1.65-8.34) in Korean men [8]. Although that study suggested an association between the haplogroup that includes mtDNA SNP (m.1382A>C) at the MOTS-c region and T2D, the relation between the m.1382A>C polymorphism and human T2D pathophysiology was not explored. Additionally, whether MOTS-c and its K14Q variant are associated with disease risk or have a direct physiological effect on insulin and glucose metabolism or the pathogenesis of T2D have not yet been investigated.

Here, we elucidated the association between T2D and m.1382A>C in Japanese individuals and established the abnormal biological effects of the K14Q MOTS-c peptide variant – a consequence of the m.1382A>C polymorphism – on insulin action and adiposity *in vitro* and *in vivo*. Additionally, it has been suggested that the mitochondrial haplogroup D4b2 is associated with exceptional longevity according to a previous study with a small sample size (n = 96) of centenarians [23]. But, as shown below, our expanded data (n = 736) indicates that the m.1382A>C polymorphism associated with the K14Q MOTS-c variant does not affect lifespan.

## RESULTS

### WT and K14Q MOTS-c potentially have different structure

The m.1382A>C polymorphism (rs111033358) is located in the 12S rRNA within the mtDNA, which encodes the ORF for the MOTS-c peptide. The m.1382A>C polymorphism could alter both the 12S rRNA structure and causes a K14Q replacement in the MOTS-c peptide. We first predicted the 12S rRNA secondary structure by m.1382A and m.1382C and found very little difference between the two models (Supplementary Figure 1A). We next examined the difference between WT MOTS-c (K14) and K14Q MOTS-c (Q14) peptides. The electrostatic potential map of the MOTS-c and the K14Q MOTS-c reflect the amino acid change from a positively charged lysine to a neutral glutamine at the 14^th^ residue of WT MOTS-c (Supplementary Figure 1B). As expected, the net charge of K14Q MOTS-c at pH7.0 was lower than WT MOTS-c and the hydrophobicity increased compared to WT MOTS-c (Supplementary Figure 1C). We also utilized a bioinformatics prediction tool called mPROVEAN (PROtein Variation Effect Analyzer; http://provean.jcvi.org), that predicts the functional effects of protein sequence variations, to predict whether single amino acid substitutions in the MOTS-c region could be detrimental or neutral [24]. A PROVEAN score was generated for the m.1382A>C polymorphism on MOTS-c that makes K14Q MOTS-c. The PROVEAN score for MOTS-c K14Q replacement was -4.0 which is below the predicted cutoff score (= -2.5). If the PROVEAN score is smaller than or equal to a given threshold, the variation is predicted as deleterious. Thus, the result suggests that K14Q MOTS-c replacement could be detrimental [24]. We next compared the predicted structures of WT and K14Q MOTS-c which were predicted via the de novo modeling servers, PEP-FOLD3 and I-TASSER (Model 1, Supplementary Figure 1D, 1E; Model 2, Supplementary Figure 1F, 1G). The predicted 3D structure of WT and K14Q MOTS-c showed a mainly loop structure and a small helix at the N-terminus, suggesting a flexible conformation for the peptides. Thus, the primary impact of the K14Q mutation may be the reduction of the positive charge of the peptide which could substantially alter its molecular interactions with its binding partners and the related biological function.

### Insulin sensitizing effects of WT and K14Q MOTS-c *in vitro*

To test the possibility that the altered structure of MOTS-c resulting from the K14Q mutation will lead to reduced activity of the peptide, we employed both forms of MOTS-c in several *in vitro* models. Since MOTS-c has been shown to increase insulin sensitivity in L6 myotubes and mouse skeletal muscle via glucose clamps and insulin action assays, we assessed the effect of K14Q MOTS-c on cellular insulin action by exogenous peptide administration and by transient transfection.

**Figure 1:**
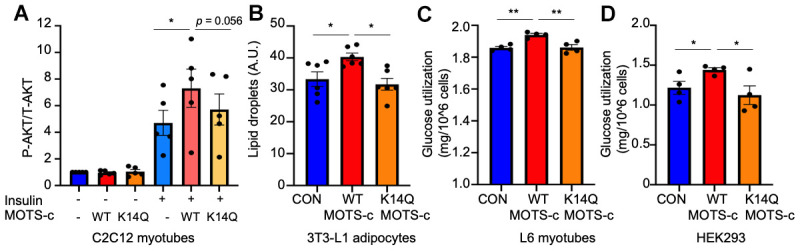
**K14Q MOTS-c has a reduced insulin sensitizing effect compared to WT MOTS-c in muscle and fat cell lines.** (**A**) Differentiated C2C12 myotubes, were treated with WT and K14Q MOTS-c in the absence or presence of 10-nM insulin. The total and phosphorylated AKT (Ser473) levels were measured by MSD. (**B**) 3T3L1 Cells were incubated with 1-μg/mL insulin +/- WT MOTS-c or K14Q MOTS-c. At day 12, lipid droplets were measured by Nile Red staining. (**C**) Differentiated L6 myotubes were treated with WT and K14Q MOTS-c for 96hr. (**D**) Overexpression of WT MOTS-c, but not K14Q MOTS-c, increases glucose uptake in HEK293 Human embryonic kidney cells. HEK293 cells were transiently transfected with WT MOTS-c and K14Q MOTS-c for 72 hr. Glucose levels in the medium were measured. Glucose utilization was calculated by [Total amount of glucose in the media] – [remaining glucose in the media]. Data are reported as mean ± SEM of seven independent experiments. *** *p* < 0.001** *p* < 0.01, *p < 0.05.

To examine the insulin sensitizing effect of MOTS-c and its variant, we treated cells with insulin in the absence or presence of WT or K14Q MOTS-c in differentiated C2C12 myotubes. Upon insulin stimulation, insulin receptor tyrosine kinase activates AKT via PI3K. AKT phosphorylation by insulin is higher in WT MOTS-c than in the control and in K14Q MOTS-c treatment in differentiated C2C12 myotubes (Figure 1A). WT MOTS-c further increased AKT phosphorylation in the presence of insulin, whereas K14Q MOTS-c showed a blunted response to insulin-stimulated AKT phosphorylation in the presence of insulin in differentiated C2C12 myotubes (Figure 1A). In addition, insulin plays an important role in adipocyte differentiation. Hence, we used 3T3-L1 pre-adipocyte differentiation as another model to assess insulin sensitivity. During 3T3-L1 pre-adipocyte differentiation, cells were incubated with insulin (1-μg/mL) containing medium. We treated cells with insulin (0, 0.5, and 1-μg/mL) in the presence or absence of WT MOTS-c. MOTS-c increased lipid droplets only in the presence of insulin, suggesting MOTS-c has an insulin sensitizing effect on adipocyte differentiation (*data not shown*). Next, we treated cells with WT and K14Q MOTS-c in the presence of 1μg/mL insulin. WT MOTS-c, but not K14Q MOTS-c increased lipid droplets during adipocyte differentiation (Figure 1B). Next, differentiated L6 myotubes were treated with WT and K14Q MOTS-c. Glucose levels were measured in the conditioned medium. WT MOTS-c increased glucose uptake, whereas K14Q MOTS-c was less effective at enhancing insulin-stimulated glucose uptake than WT MOTS-c (Figure 1C). We also transiently transfected HEK293 cells with WT MOTS-c and K14Q MOTS-c and measured glucose uptake. WT MOTS-c increased glucose uptake, whereas K14Q MOTS-c effects were the same as the control plasmid (Figure 1D). Taken together, our *in vitro* studies show that K14Q MOTS-c has a dramatically diminished action as an insulin sensitizer compared to WT MOTS-c.

### WT MOTS-c and K14Q MOTS-c have differential effects on male mice fed a high fat diet

**Figure 2:**
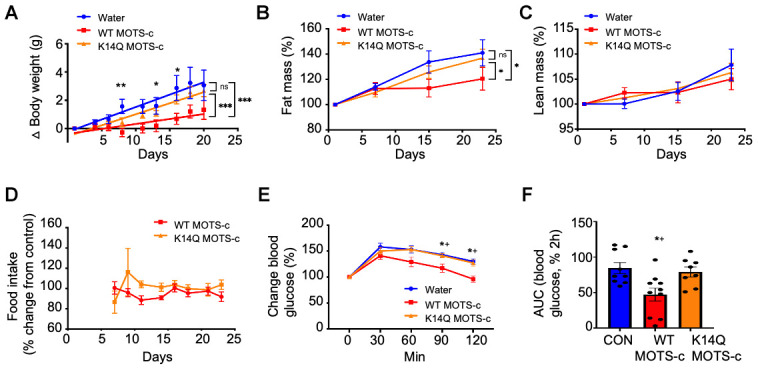
**K14Q MOTS-c is less effective than WT MOTS-c, on reducing body weight, fat mass, and glucose tolerance in CD1 male mice exposed to high-fat diet.** (**A**–**D**) Male CD-1 mice (14 weeks old) fed a high-fat diet (HFD, 60% by calories) (n = 8-12) treated with WT and K14Q MOTS-c (7.5 mg/kg; IP; BID) for 21 days. (**A**) body weight, (**B**) fat mass, (**C**) lean mass, (**D**) food intake. (**E**, **F**) Eight-weeks old male CD-1 mice fed a HFD diet (n = 8-10) treated with WT and K14Q MOTS-c (0.5 mg/kg; IP; daily) for 21 days, at which point they were assessed with a glucose tolerance test (GTT). Shown are: (**E**) Blood glucose and (**F**) glucose AUC, during the GTT. (**A**–**D**) *** *p* < 0.001, ** *p* < 0.01, *p < 0.05. (**E**, **F**) *p < 0.01 Water vs. WT MOTS-c. †*p* < 0.01, K14Q MOTS- vs. WT MOTS-c.

As MOTS-c has beneficial effects on metabolism and acts as an exercise mimetic, we speculated that individuals who carry the C allele, which produces the K14Q MOTS-c peptide will have a reduced insulin-sensitizing effect compared to WT MOTS-c in animal models. Previous studies of exogenous MOTS-c treatment in mice showed a substantial effect of improving insulin action and since peptide treatments are clinically translatable, we treated mice with exogenous WT- and K14Q MOTS-c. We investigated the effect of WT and K14Q MOTS-c in a diet-induced obesity mice model. We administered WT and K14Q MOTS-c (7.5 mg/kg; twice daily (BID); IP) to CD1 male mice fed a high-fat diet (HFD, 60% by calories) to examine the roles of K14Q MOTS-c in diet-induced metabolic alterations. Our previous studies showed that MOTS-c reduced body weight gain and fat mass, improved insulin sensitivity in skeletal muscle, and heightened energy expenditure in HFD-fed CD1 male mice. In this study, we confirmed our previous findings showing that WT MOTS-c significantly reduced weight gain in high-fat diet-fed male mice (Figure 2A). In contrast to WT MOTS-c, K14Q MOTS-c failed to protect against HFD-induced weight gain, showing no effect *in vivo*; total body weight was identical between K14Q MOTS-c and water-treated controls (Figure 2A). Moreover, HFD feeding induced a similar fat-weight increase in K14Q MOTS-c and water-treated control, whereas WT MOTS-c showed a reduction in fat-weight compared to controls after 3 weeks of HFD consumption (Figure 2B). There was no effect on lean mass in the HFD-fed mice treated with either WT or K14Q MOTS-c (Figure 2C). Although body weight was lower in the WT MOTS-c treated group, food intake was identical between the three groups (Figure 2D). Since MOTS-c increased cellular glucose uptake and increased insulin sensitivity *in vitro,* we hypothesized its actions *in vivo* would be related to glucose clearance indicative of improved insulin sensitivity. We treated mice with WT MOTS-c and K14Q MOTS-c for 21 days and then subjected them to a glucose tolerance test (GTT). We observed that WT MOTS-c significantly enhanced glucose clearance, whereas the glucose tolerance curves were not different between K14Q MOTS-c and water-treated group (Figure 2E, 2F). Taken together, K14Q MOTS-c is a far less effective metabolic regulator in male mice *in vivo* compared to WT MOTS-c (showing no discernable effects at the doses tested here).

### WT MOTS-c and K14Q MOTS-c lacked effects on female mice fed a high fat diet

**Figure 3:**
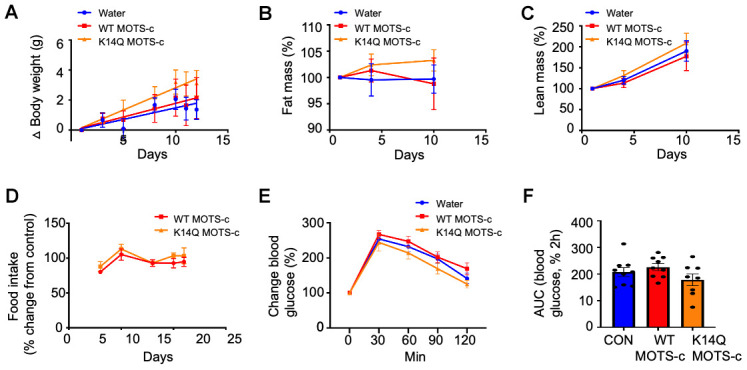
**Lack of a MOTS-c effect in female mice.** 12 weeks old CD1 female mice fed a high fat diet were treated with WT MOTS-c and K14Q MOTS-c similarly to Figure 2A–2D (n = 8) for 12 days. (**A**) body weight, (**B**) fat mass, (**C**) lean mass, (**D**) food intake. Eight-weeks old female mice fed a HFD diet (n = 8-10) treated with WT and K14Q MOTS-c (0.5 mg/kg; IP; daily) for 21 days and assessed by a glucose tolerance test (GTT). Shown are (**E**) Blood glucose and (**F**) glucose AUC, during the GTT.

To identify potential sex difference in MOTS-c effects in mice, we administrated WT and K14Q MOTS-c (7.5 mg/kg; BID; IP) in CD1 female mice fed a HFD (60% by calories). Unlike male mice, female mice did not respond to WT MOTS-c treatment (Figure 3A). Female mice injected with K14Q MOTS-c showed no effect either (Figure 3A). Fat mass, lean mass, and food intake were also not altered in WT or K14Q MOTS-c-injected female mice (Figure 3B–3D). We also administrated female mice with WT MOTS-c and K14Q MOTS-c for 21 days and then subjected them to a glucose tolerance test (GTT). We observed that glucose curves for both WT MOTS-c and K14Q MOTS-c were not different from controls (Figure 3E, 3F).

### The association between visceral fat and the MOTS-c polymorphism

**Figure 4:**
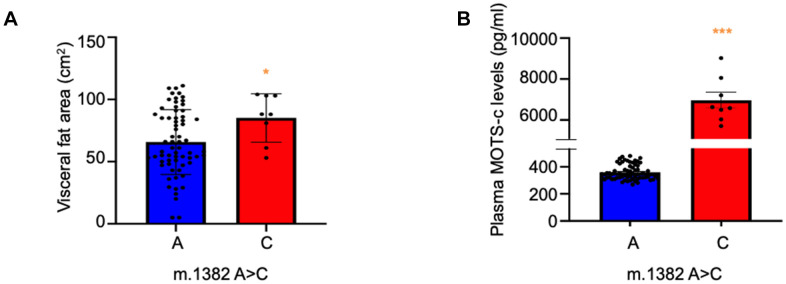
**Increased visceral fat mass and MOTS-c levels in m.1382A>C polymorphism carriers.** (**A**) Visceral fat and (**B**) Plasma MOTS-c levels were measured in individuals carrying either reference (m.1382 A) or alternative (m.1382 C) alleles. n=75, *** *p* < 0.001, *p < 0.05.

The causes and pathophysiology of T2D differs in East Asians compared to Caucasians. Asian diabetic subjects typically present with a lower BMI, but with more visceral fat; they have a younger average age of diabetes onset and display greater initial pancreatic beta cell dysfunction [5]. To assess differences between non-diabetic K14Q MOTS-c SNP carriers and controls, we evaluated the Tsukuba cohort of 73 men (67 m.1382A and 8 m.1382C). We hypothesized that the production of K14Q MOTS-c, that is far less effective in reducing fat in mice compared to WT MOTS-c, may be associated with altered body composition. Importantly, using dual-impedance analysis, we found that MOTS-c SNP carriers exhibited significantly higher visceral fat compared to controls matched for age and BMI (p < 0.05, Figure 4A). This result suggests that the MOTS-c polymorphism could lead to abdominal obesity and participate in the pathogenesis of T2D in East Asians.

### m.1382A>C polymorphism is associated with higher circulating MOTS-c levels

K14Q MOTS-c has reduced activity in cellular and animal models, compatible with this version of the peptide being partially bioinactive. In many human syndromes associated with mutations in peptide hormones, the relative deficiency of the activity of the hormone leads to increased production and higher levels of such hormones [25–29]. Thus, we examined the effect of the MOTS-c polymorphism on circulating MOTS-c levels. We measured plasma levels of MOTS-c WT and SNP carriers by ELISA in the subjects from the Tsukuba cohort. Compared to age-, weight-, and gender-matched controls, m.1382A>C carriers displayed dramatically (nearly 20-fold) higher MOTS-c levels (p < 0.0001; Figure 4B). To understand whether the higher levels of K14Q MOTS-c are due to higher production rates or slower clearance rates, we infused both WT and K14Q MOTS-c into mice using intraperitoneal osmotic pumps and measured steady-state plasma levels of the peptides by ELISA. Clearance rates and production rates were calculated as we previously described [30, 31]. The clearance rate of K14Q MOTS-c was 2.6 times slower than that of WT, but the production rate of K14Q MOTS-c was calculated to be 7.4 times higher than that of WT. These results suggest that MOTS-c production in subjects with the m.1382A>C mutation are increased in an attempt to compensate for the reduced biological activity of the K14Q MOTS-c variant.

### The m.1382A>C MOTS-c polymorphism affects T2D incidence in Japanese men

**Figure 5:**
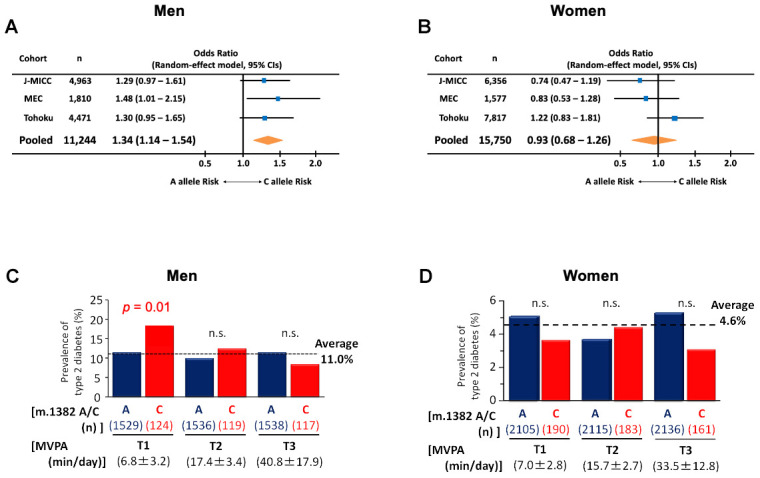
**The prevalence of type 2 diabetes in m.1382A>C polymorphism carriers.** (**A**) Forest plot for meta-analysis (pooled random effects) on the prevalence of type 2 diabetes in males. Odds ratios are adjusted by age and BMI. Test for the pooled effect, Z = 2.86, *p* < 0.01. Heterogeneity was insignificant (I^2^ = 0%, *p* = 0.84). (**B**) Forest plot for meta-analysis (pooled random effects) on the prevalence of type 2 diabetes in female. Odds ratios are adjusted by age and BMI. Test for the pooled effect, Z = -0.46, *p* = 0.64. Heterogeneity was insignificant (I^2^ = 33.6%, *p* = 0.22). In Japan Multi-Institutional Collaborative Cohort (J-MICC) study, m.1382A>C polymorphism carriers divided by tertile of physical activity in (**C**) males and (**D**) females. Activity was assessed by the degree of moderate-to-vigorous intensity physical activity (MVPA). In men only, sedentary levels of MVPA were associated with an increased risk of diabetes in the C allele. Cochran-Armitage trend test of m.1382 C allele for men, χ^2^ = 6.26, *p* = 0.012. Cochran-Armitage trend test of m.1382 A allele for females, χ^2^ = 0.03, *p* = 0.861.

So far, we showed K14Q MOTS-c produced from individuals carrying m.1382C is less effective as a metabolic regulator *in vitro* and *in vivo* and is associated with elevated hormone levels and altered body composition. We further examined the effect of the m.1382A>C polymorphism on T2D in three additional cohorts of individuals of Japanese descent. First, we used the Japan Multi-Institutional Collaborative Cohort (J-MICC) Study, which includes 4,963 men and 6,889 women, of whom 98.2% had documented BMI, smoking status, diagnosis of T2D, m.1382A>C polymorphism status (Supplementary Table 1). Second, we used Japanese-American subjects in the Multiethnic Cohort (MEC) study, which includes 1,810 men and 1,577 women of Japanese descent living in the US. Third, we used the Tohoku Medical Megabank project (TMM), which includes 4,471 males and 7,817 females (Supplementary Table 2). Meta-analysis of the three cohorts showed that the m.1382A>C polymorphism significantly increases the prevalence of T2D (*p* < 0.01) in males but not in females (Figure 5A, 5B). Furthermore, in the MEC study, men with the C allele showed increase in T2D risk (*p* = 0.04) compared to A allele carriers (Supplementary Table 3).

### A Kinesio-Genomic effect of a MOTS-c SNP on T2D incidence in men

As MOTS-c has been suggested to interact with exercise [32], we took advantage of the accelerometer-based measurement of daily activity in the J-MICC study to examine if the amount of exercise contributes to diabetes risk in SNP carriers. Surprisingly, the m.1382A>C polymorphism increases T2D risk mainly in sedentary males in J-MICC. Men with the C allele of the m.1382A>C polymorphism showed a trend for higher risk for T2D regardless of physical activity status measured by accelerometer (C allele, 13.1%; A allele, 10.8%; *p* = 0.196, Supplementary Table 1 top), however, when assessing potential contributors to T2D risk, the degree of physical activity, measured by accelerometer, and expressed as moderate- to vigorous-intensity physical activity (MVPA), correlated with lower risk for T2D in men with the C allele (MVPA beta = 0.023; *p* = 0.035; Supplementary Tables 4, 5). As shown in Figure 5C, men with the C allele and low physical activity (T1 of MVPA: 6.8 ± 3.2 min/day) had a 65% greater rate of T2D than men with the A allele and low physical activity (A allele T2D prevalence = 11.2%; C allele T2D prevalence = 18.5%; *p* = 0.014; Figure 5C). This relationship is not seen in men with higher physical activity. The effect of m.1382A>C is male-specific, as females with the C allele who were classified as sedentary (physical activity T1 = 7.0 ± 2.8 min/day) did not have an increased T2D risk (A allele T2D prevalence = 5.1%; C allele T2D prevalence = 3.7%; *p* = 0.258; Figure 5D). No significant differences were found for other characteristics between the m.1382A>C genotype in men with the lowest MVPA (Supplementary Table 6). These results strongly suggest that a combination of sedentary lifestyle and the m.1382A>C polymorphism contributes to elevated T2D risk. Unfortunately, the TMM study and MEC study only quantified physical activity using a questionnaire, which is different from the accelerometer-based measurement used by J-MICC. Physical activity questionnaires do not correlate well with accelerometer-based measurements in young and old individuals [33–36]. Hence, we are unable to assess physical activity behavior among the three cohorts using a harmonized approach (i.e., accelerometer). As a consequence, it appears that the kinesio-genomic relationship of m.1382A>C genotype and T2D can only be teased out with accurate exercise assessment in the J-MICC cohort.

### Effects of other polymorphisms on the incidence of T2D

We assessed the specific effect of the m.1382A>C polymorphism on T2D risk to show that it is independent from the other polymorphisms that are considered determining for haplogroups D, D4 and D4b (that are associated with diabetes risk) by using J-MICC cohort. The m.1382A>C polymorphism is one of the determining polymorphisms in the mitochondrial haplogroup D4b2. This haplogroup is most frequent among East Asians including Japanese, Koreans, and Northern Chinese. Korean men in haplogroup D4b have previously been shown to have a dramatically increased risk of T2D (OR 3.55 [95% CI 1.65–8.34], *p* = 0.0019) [8]. We analyzed four additional SNPs defining haplogroup D: m.4883C>T (ND2: syn), m.5178C>A (ND2: L237M), haplogroup D4: m.3010G>A (16S rRNA), and haplogroup D4b: m.15440T>C (CYB: D4b1) and m.1382A>C (D4b2) for an effect on T2D risk (Supplementary Tables 7–10). None of these SNPs were associated with T2D risk; only m.1382A>C significantly increased T2D prevalence in sedentary male subjects (Supplementary Table 11). We did not look at the effect of m. 8020G>A (D4b) because: 1) m. 8020G>A status was not collected in J-MICC; and 2) The polymorphism m.8020G>A is not only a SNP of haplogroup D4b but also a SNP of other haplogroups (i.e., M7b1a1a1a, D6a1a, R0a1a2, H5k, J1c1g1, F2e1, F4b and K1a14). The D4b haplogroup (defined as possessing both the m.15440 and m.1382 polymorphisms) and the D4b1 determinant SNP, m.15440, also do not show any effect on the risk of T2D (Supplementary Table 10). A previous study reported that haplogroup D4b was associated with T2D in Korean men, but not women [8]. We hypothesized that the increase of risk in Korean male carriers of haplogroup D4b is driven by the m.1382A>C polymorphism. Because m.1382A>C defining sub-haplogroup D4b2 is a major component of haplogroup D4b, it is likely that this polymorphism influences the increased risk in T2D in Korean men due to the resultant K14Q MOTS-c variant. We speculate that the increased risk in Korean male carriers of haplogroup D4b is driven by the m.1382A>C polymorphism because the D4b haplogroup itself does not impact T2D risk in J-MICC study.

### Effect of K14Q MOTS-c (m.1382A>C) polymorphism on longevity

m.1382A>C polymorphism is very common in the mitochondrial haplogroup D4b2. Interestingly, haplogroup D4b2 was reported to be associated with exceptional longevity, albeit with small sample size of 96 centenarians, [22, 23] and the m.1382A>C polymorphism was speculated to be involved in the extended lifespan of individuals harboring this haplogroup. To further assess this possibility, we used an expanded cohort of Japanese centenarians (n = 736) and analyzed the allele frequency of the m.1382A>C variant to specifically decipher the potential involvement of this SNP in lifespan. Allele frequency was similar between centenarian and control groups. C allele frequencies in centenarians and controls were 7.7% (8.4% in men and 7.6% in women) and 7.5% (7.3% in men and 7.8% in women), respectively. Taken together, we concluded m.1382A>C polymorphism is unlikely to be involved with exceptional longevity.

### WT MOTS-c and K14Q MOTS-c have no effect on voluntary physical activity

We showed that K14Q MOTS-c has a reduced effect on insulin sensitivity and weight gain compared to WT MOTS-c in mice and speculate that the m.1382A>C polymorphism increases the prevalence of T2D in sedentary men because the C allele carriers produce a less effective K14Q MOTS-c. However, an alternative explanation for the increased T2D frequency is that the m.1382A>C polymorphism can impact voluntary physical activity, which in turn affects T2D prevalence (although the frequency of the C allele was not statistically different between the tertile of physical activity in the J-MICC study as shown in Supplementary Table 4). We analyzed a voluntary wheel running activity for five days in male and female mice injected with WT MOTS-c and K14Q MOTS-c (7.5-mg/kg; BID; IP). Neither WT MOTS-c or K14Q MOTS-c had a significant effect on voluntary physical activity (Supplementary Figure 3A–3F). Therefore, we ruled out the possibility that voluntary physical activity plays role in the m.1382A>C polymorphism (and MOTS-c variation) effect on T2D prevalence.

## DISCUSSION

We found that the C allele of the m.1382A>C polymorphism in the mtDNA leads to the expression of an altered mitochondrial-derived peptide, K14Q MOTS-c, which is associated with susceptibility to T2D and increased visceral fat in men. The K14Q MOTS-c peptide is less effective in promoting insulin sensitization and weight loss than wild type MOTS-c *in vitro* and *in vivo* studies. We also identified a sex difference in terms of the effects of the m.1382A>C polymorphism on T2D risk in humans and a related difference in the response to peptide administration in terms of glucose tolerance and weight gain in response to HFD feeding in mice. These observations represent a male-specific phenomenon. We also noted that the levels of MVPA are related to the prevalence of T2D in C allele carrying men, representing a novel kinesio-genomic interaction. These results provide us a better understanding of the contribution of mitochondrial genetic variants in biological pathways of diseases and insights on therapeutic targets in treating T2D based on personalized medicine in East Asia.

K14Q MOTS-c represents a partially bioinactive form of WT MOTS-c. The circulating levels of MOTS-c, as measured by ELISA, are dramatically elevated in subjects carrying the m.1382C allele, supporting a compensatory attempt to respond to the mutation. This type of phenomenon is typical in conditions where mutations in genes of various hormones lead to the compensatory over-production of bioinactive forms of these hormones that have been shown to be associated with decreased activity at the receptor levels. Examples for this include patients with short stature with IGF-1 gene mutations that produce bioinactive IGF-1 who display elevated immunoreactive IGF-1 levels [37], short patients with mutant GH gene and high levels of measured growth hormone which is bioinactive [25], as well as patients with glucocorticoid deficiency caused by a mutation leading to bioinactive ACTH and elevated immunoreactive ACTH levels [26]. Similarly, TSH mutations reducing the affinity of the hormone to its receptor and causing hypothyroidism [27], bioinactive leptin associated with obesity [28], and bioinactive insulin leading to MODY diabetes [29] have all been described. In all these cases, the over-secretion of the completely or partially bioinactive peptide hormone that is unable to fully compensate is eventually associated with a state resembling a hormone deficiency. In the case of bioinactive MOTS-c, our data clearly shows that the K14Q variant is bioinactive in some *in vitro* assays and does not elicit the normal biological effect *in vivo*. It is rational to assume that Japanese males with the MOTS-c mutation that leads to a bioinactive form of this metabolic regulator can compensate through certain mechanisms including the insulin sensitization resulting from exercise, but in some subjects, this compensation eventually fails, leading to increased diabetes rates, especially in combination with sedentary lifestyle.

The results suggest that higher MVPA rescues the potentially deleterious effects of the m.1382A>C polymorphism on T2D risk in men. Exercise could be an especially important beneficial intervention for C allele carriers of this SNP. Developing optimized exercise guidance for people with the m.1382A>C polymorphism could be clinically translatable. It is compelling to hypothesize that MOTS-c is an exercise mimetic peptide that helps prevent T2D in sedentary men while in men who are frequent exercisers there is no further metabolic benefit of MOTS-c, or further protection from T2D by MOTS-c. However, the bioinactive K14Q MOTS-c may increase T2D risk, particularly in the susceptible sedentary group. We propose the concept that some variants of mtDNA evolved to best fit living conditions characterized by highly active lifestyles and limited caloric intake, where K14Q MOTS-c, which might be less effective in fast-to-slow type muscle fiber transition and increase fast type fiber [18], might present an advantage, but in the twenty-first century, it is a metabolic liability.

Our data shows that the effect of K14Q variant on T2D is male-specific. The mother-to-son transmission of an unfavorable mitochondrial genetic trait has been previously described and named the “mother’s curse” [38]. Hormonal differences between males and females could interact differentially with MOTS-c on metabolic function [39]. MOTS-c was shown to prevent ovariectomy-induced metabolic dysfunction. MOTS-c did not change the metabolic phenotype in female mice with normal hormonal function (similar to our finding here), but MOTS-c improves metabolic function in ovariectomized female mice [20]. These results suggest a link between ovarian hormones and the effect of MOTS-c. Thus, it could be clinically relevant to identify what sex-specific factors permit females to overcome the potentially harmful effect of the m.1382A>C polymorphism on T2D seen in sedentary men. These may include sex-specific variations in mitochondrial mass or function or direct effects of sex-steroids on mitochondrial biology that favor females [40].

We believe these results are generalizable to the entire Japanese population and likely to many other East Asians. The prevalence of T2D in the present study was similar to the general Japanese population in their 50’s (men = 11.7%; women = 5.5%) [41], and the subject’s characteristics are representative of the mean BMI of the Japanese population in their late 50’s (men = 23.6 ± 1.3 kg/m^2^; women = 21.7 ± 1.1 kg/m^2^) [42].

The East Asian populations that may carry the K14Q MOTS-c SNP include around half a billion people in which the frequency of the m.1382A>C polymorphism varies from 5 to 8%. Sedentary behavior is alarmingly common in modern Asian cities [43]. The reported rate of diabetes in East Asian countries is well over 10%, and rising rapidly [44], suggesting that several million men with T2D carry the kinesio-genomic MOTS-c SNP. As MOTS-c analogues are currently in clinical development for the treatment of T2D complications, including nonalcoholic steatohepatitis, the recognition of the m.1382A>C associated kinesio-genomic T2D risk may inform future clinical trials. This novel discovery suggests that additional ethnic-specific mtDNA polymorphisms, that might affect the structure or expression of MOTS-c or other MDPs, may be involved in metabolic disease risk.

In summary, we found that the C allele of the m.1382A>C polymorphism changes body composition and increases the risk of T2D in Japanese men, especially in sedentary individuals. This polymorphism causes an amino acid replacement from Lys (K) to Gln (Q) at amino acid 14 in the MOTS-c peptide, which renders it a less potent insulin-sensitizer compared to WT MOTS-c. A deeper understanding the effects of this genetic polymorphism will provide a basis for developing physical activity strategies to maximize the benefits of exercise in T2D.

## MATERIALS AND METHODS

### Epidemiological study

### Japan multi-institutional collaborative cohort (J-MICC)

A cohort in which the relationship between genes and lifestyle was examined [45]. This cross-sectional study consisted of 12,068 subjects in Saga City (men, 5,078; women, 6,990) who were between 40–69 years old. The Saga J-MICC Study was approved by the ethics committees of both the Saga University Faculty of Medicine and Nagoya University Graduate School of Medicine. The study conformed to the principles outlined in the Declaration of Helsinki. Written informed consent was obtained from all subjects before their inclusion in the study. A variety of lifestyle measurements were recorded. The baseline survey was conducted from November 1, 2005 through December 22, 2007 [46]. A self-administered questionnaire was used to collect data on smoking, dietary habits, current medication, disease history, and family history. Daily physical activity was objectively measured using an accelerometer (Life-Corder; Suzuken, Nagoya, Japan) as previously described [47]. Height and weight were measured to the nearest 0.1 cm and 0.1 kg, respectively. Body mass index (BMI) was calculated as the weight in kilograms divided by the square of the height in meters (kg/m^2^). Waist circumference was measured to the nearest 0.1 cm at the midpoint between the lower costal margin and the iliac crest using a calibrated measuring tape. The HbA_1c_ (%) level was measured and converted from the Japan Diabetes Society (JDS) to the National Glyco-hemoglobin Standardization Program (NGSP) by using the following equation published by the JDS: NGSP (%) = 1.02 × JDS (%) + 0.25% [48]. T2D in subjects was defined as either a positive response to a questionnaire, prescription of a diabetes medication, or an HbA_1c_ over 6.5%. Mitochondrial genetic variants were captured as described previously [8, 49]. Briefly, mitochondrial polymorphisms were determined with sequence-specific oligonucleotide probes (G&G Science, Fukushima, Japan) by use of suspension array technology (Luminex 100).

### Multiethnic cohort (MEC)

MEC is a population-based prospective cohort study including approximately 215,000 men and women from Hawaii and California that is part of the Population Architecture using Genomics and Epidemiology (PAGE) study. All participants were 45-75 years of age at baseline, and primarily of 5 ancestries: Japanese Americans, African Americans, European Americans, Hispanic/Latinos, and Native Hawaiians. All cohort members completed baseline and follow-up questionnaires. For the current study, MEC contributed data from Japanese Americans with T2D and Japanese American case-control subjects (1,799 cases and 1,588 controls), both men and women with complete covariate data. Single variant association testing was completed among 3,387 MEC Japanese Americans with complete genotype data for the m.1382A>C variant (rs111033358). A logistic regression model adjusted for age and BMI was used to identify stratified gender specific associations. All analyses were performed in SAS and adjusted beta values, odds ratios, 95% confidence intervals and p-values were reported.

### Tohoku medical megabank project (TMM)

Over 80,000 apparently healthy adults living in the Pacific coast of the Tohoku region in Japan were recruited from May 2013 to March 2016 for the TMM as previously described [50]. Approval was obtained from the relevant ethics committees. All participants gave written informed consent at the time of study enrolment. A total of 9,559 participants were genotyped using the HumanOmniExpressExome BeadChip Array (Illumina Inc., San Diego, CA, USA). 3,552 participants were genotyped by whole genome sequencing using HiSeq 2500 system. Subjects reported histories of medical conditions, including that of T2D, in a research questionnaire. Association between mtDNA 1382A>C and T2D was tested by linear regression analysis using R version 3.5.1.

### Centenarian cohort

This cohort (subjects aged 100 years or older) consisted of 743 Japanese centenarians (119 men; 617 women) who were registered by the Center for Supercentenarian Medical Research of Keio University School of Medicine. The study was approved by the ethics committee of Keio University School of Medicine and written informed consent was obtained from all individuals. The m.1382A>C polymorphism (rs111033358) was analyzed by using custom TaqMan SNP Genotyping Assay. An allele frequency of m.1382A>C variant in the J-MICC cohort (40-69 years old) was used as control. Statistical analyses were performed using JMP Pro version 12 (SAS Institute).

### Tsukuba male cohort

This study was approved by the ethical committee of the Faculty of Medicine at the University of Tsukuba, conformed to the principles outlined in the Helsinki Declaration, and all participants were provided written informed consent before inclusion. Participants were recruited through local newspaper advertisements and personal contacts around Tsukuba-city in Japan. A total of 75 subjects (age range: 43-80 years old) were included in the analyses. Visceral fat area was measured using the dual-impedance analysis method (HSD-2000; Omron Healthcare, Kyoto, Japan) [51]. Blood samples were taken in the morning after 12-h overnight fast. Total DNA was isolated form plasma samples by using QIAamp DNA Blood Mini Kit (Qiagen, Hilden, Germany), according to the manufacturer’s instructions. m.1382A>C (rs111033358) polymorphism was analyzed by digital droplet PCR mutation detection assays according to the manufacturer’s instruction (Biorad, Cat#10049047).

### Meta-analysis

We performed a meta-analysis to compare the prevalence of T2D between m.1382A and C allele to pool the results from the three cohorts (J-MICC, MEC, and TMM). Meta-analysis was conducted using the R package “metafor”. The subjects of analysis have all the data. The DerSimonian and Laird random-effects model were used, and analysis was performed with odds ratio adjusted by age and BMI that were associated with the onset of T2D. This analysis takes into account confounding factors and population heterogeneity among the cohorts (potential bias between studies). The between population heterogeneity was assessed by using the I^2^ statistic, that a higher I^2^ values mean higher heterogeneity. Significance of the pooled effect was determined by the Z test, and 95% CIs were calculated.

### Cell culture

C2C12 mouse myoblasts, L6 rat myoblasts, and 3T3-L1 mouse embryonic fibroblasts were purchased from ATCC (Cat#. CRL-1772, CRL-1458, Cat#. CL-173; Manassas, VA, USA) and cultured in Dulbecco’s modified Eagle’s medium (DMEM) + 20% FBS, MEM +10% FBS, and DMEM + 10% Bovine Calf Serum (pre-adipocyte expansion medium), respectively (FBS; ThermoFisher Scientific, Waltham, MA, USA) at 37° C in 5% CO_2_. To differentiate C2C12 and L6 myoblasts to myotubes, media was replaced with DMEM + 2% horse serum every 24 h for 6-7 days until cells were fully differentiated. To differentiate 3T3-L1 pre-adipocytes, pre-adipocyte expansion medium was replaced with differentiation medium (DMEM,10% FBS, 1μM Dexamethasone, 0.5mM IBMX, 1μg/mL insulin) for 48 h, then replaced with adipocyte maintenance medium (DMEM 10% FBS, 1μg/mL insulin) every 2 days for 7 days. HEK293 cells were cultured in high glucose DMEM (Sigma) supplemented with 10% fetal bovine serum at 37° C in 5% CO2. For transient transfection of the wild type (WT) and K14Q MOTS-c in pcDNA3.1(+), lipofectamine 3000 was used (ThermoFisher Scientific).

### Insulin signaling

To examine insulin signaling, a fully differentiated C2C12 medium was replaced with low glucose DMEM + 0.1% fatty acid free - BSA for 2hr at 37° C. Then, the medium was replaced with PBS + 0.1% fatty acid free-BSA for 30 min and 10 nM bovine insulin was added to the cells with or without peptides for 15-min at 37° C. After the incubation, cells were washed with PBS, and protein was extracted. Total and phosphor-AKT levels were measured by MSD (MESO SCALE DISCOVERY, Rockville, MD, USA).

### Nile red staining

Seven days after 3T3-L1 cells were differentiated, lipid droplets were quantified using the Lipid Droplets Fluorescence Assay Kit (Cat#. 500001; Cayman chemical; Ann Arbor, MI, USA). Cells were plated and differentiated in a black clear-bottom 96-well plate. Cells were fixed for 10-min at RT, and stained with Nile Red staining solution for 15-min at RT. After washing the staining solution, the fluorescence intensity of lipid droplets read with filter sets designed to detect FITC (ex/em 485/535 nm).

### Measurements of glucose levels in culture media

Extracellular glucose in the cell culture medium was measured using glucose assay kits per the manufacturer’s instructions (Eton Biosciences, USA). The standard glucose solution and cell culture medium were incubated with glucose enzyme mix for 30-min at 37° C. After 30-min, the absorbance was measured on a plate spectrophotometer (Molecular Designs, Sunnyvale, CA) at 570 nm. We calculated the value of glucose in the medium using the equation obtained from the linear regression of the standard curve. The glucose utilization was calculated by [Total amount of glucose in the media] – [remaining glucose amount in the media].

### MOTS-c ELISA

WT MOTS-c levels were measured as described previously [14]. For K14Q MOTS-c measurements in plasma, we ascertained that K14Q MOTS-c could be detected by the MOTS-c ELISA albeit with less sensitivity (Supplementary Figure 2). We spiked various amount of synthetic K14Q MOTS-c peptide (4, 8, 16, 32 and 64-ng) into 100-μL human plasma samples. The spiked samples were prepared and analyzed as described above for WT MOTS-c to create a K14Q MOTS-c standard curve for the MOTS-c ELISA. To calculate K14Q MOTS-c levels in the plasma of the K14Q SNP carriers and in K14Q MOTS-c-infused mice, K14Q MOTS-c levels were calculated using this K14Q MOTS-c standard curve.

### Animals

Male and female CD-1 mice at 14 weeks (n = 8-12) and 12 weeks (n = 8-11) of age, respectively, were obtained from Jackson Laboratory (Bar Harbor, ME USA) and treated with K14Q MOTS-c and WT MOTS-c peptides. Mice were singly housed under standard 12-hr light-dark cycle with access to water and rodent food a high-fat diet (HFD, 60% by calories; Research Diets, MO). Mice were randomly assigned to one of three experimental groups: 1) a control group receiving BID (twice a day) intraperitoneal (IP) injection of vehicle (sterilized water); 2) a WT MOTS-c-treated group receiving BID IP injection of 7.5-mg per kg body weight; 3) a K14Q MOTS-c-treated group receiving BID IP injection of 7.5-mg per kg body weight. Mice were anesthetized with isoflurane and sacrificed after two weeks of treatment. Tissues were collected from the mice, flash-frozen in liquid nitrogen and stored at -80° C. To examine direct effect of K14Q MOTS-c polymorphism on T2DM in human studies, a glucose tolerance test was performed. 7-week old male (n = 8-10 for each group) and female (n = 8-9 for each group) CD-1 mice were obtained from Charles River, Japan. After a week (8-week of age), mice were singly housed under standard 12-hr light-dark cycle with access to water and rodent food a HFD (60% by calories; High Fat diet 32: CLEA, Japan). Mice were randomly assigned to one of three experimental groups 1) a control group receiving IP injection of sterilized water; 2) a WT MOTS-c-treated group receiving IP injection of 0.5 mg per kg body weight; 3) a K14Q MOTS-c-treated group receiving IP injection of 0.5mg per kg body weight, daily for 21 days. After four hours fasting from the last injection of water, WT MOTS-c, or K14Q MOTS-c (11-week of age), an intraperitoneal glucose tolerance test (GTT) was performed under isoflurane anesthesia and blood glucose was measured using a glucometer (Lab gluco, ForaCare Japan; Tokyo, Japan). GTT consisted of a D-glucose injection (1 g/kg; IP) and blood was sampled from the tail at 0, 15, 30, 45, 60, 90, and 120-min post-glucose injection. To compare WT MOTS-c and K14Q MOTS-c clearance rate in mice, we implanted ALZET^®^ osmotic pumps carrying WT MOTS-c and K14Q MOTS-c synthetic peptides into C57BL/6 mouse (15 mg/kg body weight, IP). The mice were sacrificed after 24 hours implantation and plasma samples were collected to measure steady-state WT MOTS and K14Q MOTS-c levels by MOTS-c ELISA. The peptide concentrations of the WT and K14Q MOTS-c remaining in the mini pumps after implantation were also measured. All experiments with mice were performed in accordance with the appropriate guidelines and regulations and approved by the Juntendo University and University of Southern California Institutional Animal Care and Use Committee.

### Statistical analysis

Statistical analyses were performed using PASW Statistics 18 (series of SPSS Statistics) for Windows. Differences in physical characteristics by sex were determined using unpaired t-tests and Pearson’s χ^2^ tests. The relation of the m.1382A>C genotype distribution and prevalence of T2D was evaluated using the Pearson’s χ^2^ test. Logistic regression was also used to predict the prevalence of T2D. All logistic regression models included the following covariates: age, BMI, smoking, and moderate-to-vigorous intensity physical activity (MVPA). The statistical significance for linearity between the prevalence of T2D and MVPA was evaluated using Cochran-Armitage trend tests. In animal and cell culture studies, group comparison was performed by t-test or one-way analysis of variance (ANOVA). The Bonferroni post hoc test was performed when the ANOVA indicated a significant difference. Delta body weight, fat mass, lean mass and glucose tolerance test were analyzed with two-way (group × time) ANOVA with replications. Values expressed as mean ± SD; *p* < 0.05 are considered to be statistically significant. Cell and mouse data are presented as mean ± S.E.M. Significant differences were determined by Student’s t-tests or one-way ANOVA and Turkey’s post hoc test by use of GraphPad Prism 5 software. P values of *< 0.05, **< 0.01, or ***< 0.001 were considered statistically significant.

## Supplementary Material

Supplementary Figures

Supplementary Tables
