# Supplementary material for: A pro-diabetogenic mtDNA polymorphism in the mitochondrial-derived peptide, MOTS-c
Source: Aging (Albany NY). 2021 Jan 19;13(2):1692–717. doi: 10.18632/aging.202529 (PMC7880332; doi:10.18632/aging.202529)
Supplement: Supplementary Figures [file aging-13-202529-s001.pdf]

SUPPLEMENTARY FIGURES

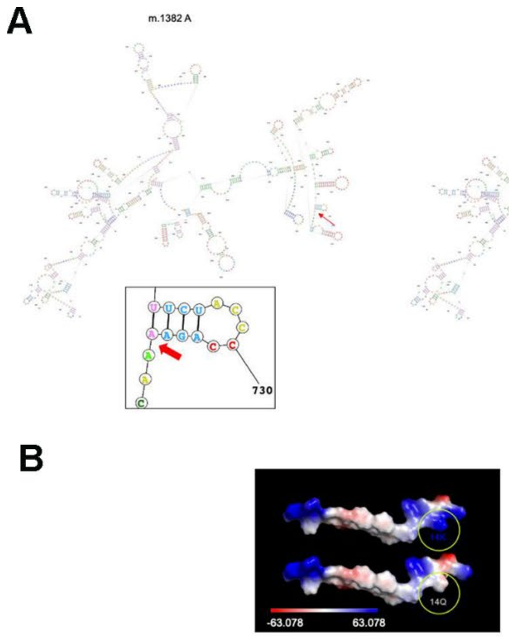

C

Theoretical physiochemical properties of MOTS-c mutant peptide.

Peptides	Molecular Weight (g/mol)	Peptide isoelectric point	Net charge at pH 7	Hydrophobicity at pH 6.8
WT MOTS-c	2174.7	10.71	+3	32.2
K14Q MOTS-c	2174.7	10.41	+2	33.0

WT MOTS-c: MRWQEMGYIFYPRKLR.

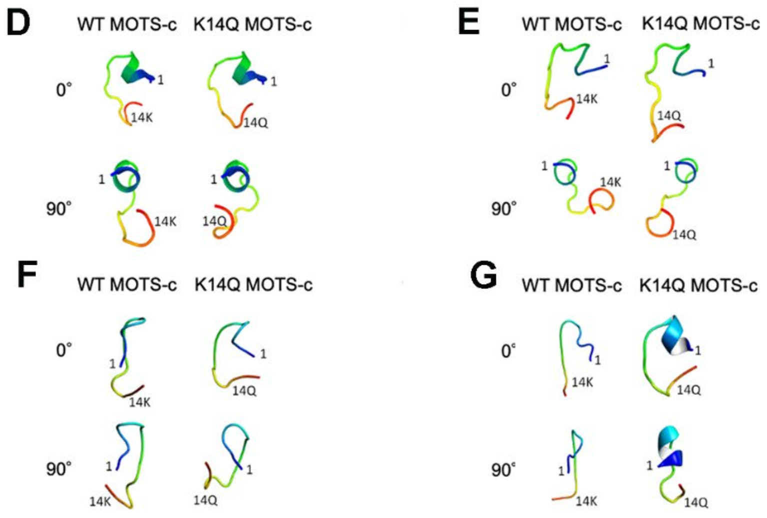

Supplementary Figure 1. The impact of m.1382 A>C polymorphism in the structure of 12S rRNA and MOTS-c peptide (**A**) The predicted 12S rRNA structure by m.1382 A>C polymorphism by RNA structure (https://rna.urmc.rochester.edu/RNAstructureWeb/Servers/Predict1/Predict1.html). The box is the enlarged view of the pointed with arrow. (**B**) The electrostatic potential map of the the MOTS-c and the K14Q MOTS-c by PyMOL. (**C**) Theoretical physiochemical properties of MOTS-c mutant peptide. (**D**, **E**) The models were obtained via the program PEP-FOLD3 (model 1). The left two structures show the model of MOTS-c 14K peptide. The right two structures show the model of MOTS-c 14Q peptide. The lower view is generated by rotating peptide structure 90° around the y-axis from upper view. (**F**, **G**) The 3D structure of MOTS-c 14K and 14Q models obtained via the program I-TASSER (model 2). The figure is generated by Molmil viewer (https://pdbj.org/molmil/).

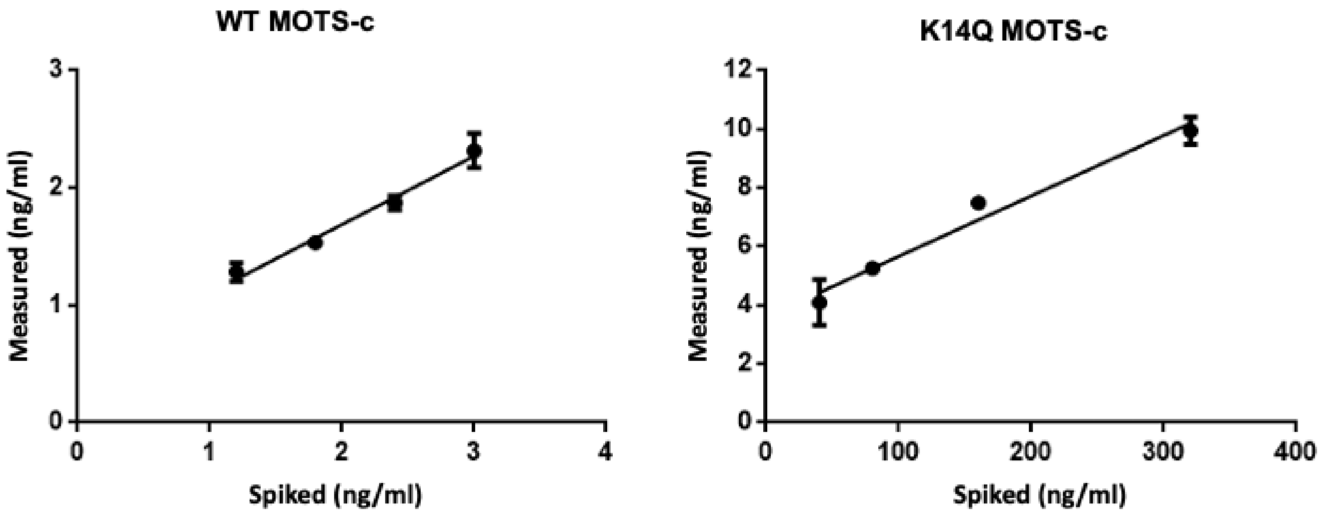

Supplementary Figure 2. Recovery of WT and K14Q MOTS-c synthetic peptides. Purchased human plasma was spiked with various concentrations of WT MOTS-c peptide (1.2, 1.8, 2.4 and 3.0 ng/ml) and K14Q MOTS-c peptide (40, 80, 160 and 320 ng/ml). Spiked WT and K14Q MOTS-c peptides were measured by MOTS-c WT ELISA described in materials and methods. Based on spiked and measured levels of MOTS-c K14Q, we calculated a conversion factor to convert MOTS-c levels to K14Q levels for human subjects carrying K14Q genotype measured by MOTS-c WT ELISA.

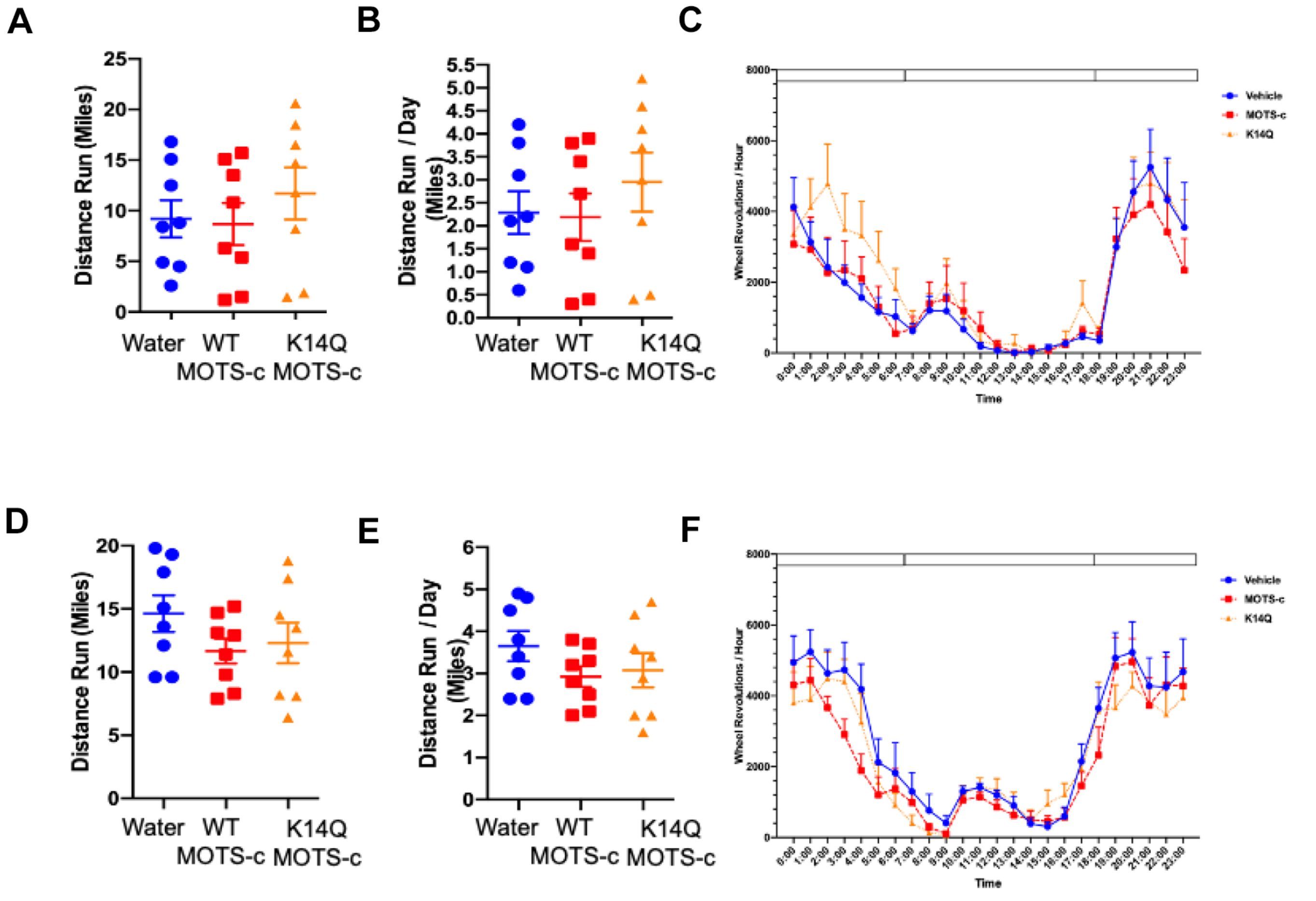

Supplementary Figure 3. The effects of MOTS-c on voluntary wheel running activity. (**A**) total distance run, (**B**) total distance run per day, and (**C**) hourly running activity in males. (**D**) total distance run, (**E**) total distance run per day, and (**F**) hourly running activity in females.
