# Supplementary material for: A pro-diabetogenic mtDNA polymorphism in the mitochondrial-derived peptide, MOTS-c
Source: Aging (Albany NY). 2021 Jan 19;13(2):1692–717. doi: 10.18632/aging.202529 (PMC7880332; doi:10.18632/aging.202529)
Supplement: Supplementary Tables [file aging-13-202529-s002.pdf]

SUPPLEMENTARY TABLES

Supplementary Table 1. Characteristics of J-MICC subjects by the m.1382A>C polymorphism.

	**A**	**C**
Male		
n	4,603	360
Age (years old)	56.6± 8.2	55.8± 8.6
Height (cm)	166.9± 6.0	167.3± 5.6
BMI (kg/m^2)	24.2± 3.0	24.3± 3.3
Waist circumference (cm)	86.2± 7.9	86± 8.7
HbA_{1c} (%)	5.3± 0.8	5.3± 0.9
Smokers (%)	35.7	38.6
Prevalence of type 2 diabetes (%)	10.8	13.1
Female		
n	6,356	534
Age (years old)	55.6± 8.2	55.8± 8.3
Height (cm)	154.5± 5.4	154.5± 5.7
BMI (kg/m^2)	22.8± 3.2	22.9± 3.4
Waist circumference (cm)	81.2± 9.4	81.4± 9.8
HbA_{1c} (%)	5.2± 0.6	5.2± 0.6
Smokers (%)	8.4	9.4
Prevalence of type 2 diabetes (%)	4.7	3.7

BMI, body mass index. Mean ± SD.

Supplementary Table 2. Characteristics of TMM subjects by the m.1382A>C polymorphism.

	A	C
Male		
n	4,199	272
Age (years old)	61.4 ± 11.5	60.8 ± 12.6
Height (cm)	162.9 ± 11.1	162 ± 12.6
BMI (kg/m2)	23.4 ± 4.4	23.4 ± 5.0
Waist circumference (cm)	83.1 ± 11.5	83.1 ± 13.0
HbA1c (%)	5.6 ± 0.7	5.6 ± 0.8
Smokers (%)	25.6	24.3
Prevalence of type 2 diabetes (%)	12.4	15.4
Female		
n	7,338	479
Age (years old)	58 ± 12.7	57.8 ± 13.1
Height (cm)	151.4 ± 8.7	150.5 ± 9.6
BMI (kg/m2)	22.4 ± 4.6	22 ± 4.9*
Waist circumference (cm)	79.5 ± 11.7	78.3 ± 12.6
HbA1c (%)	5.5 ± 0.5	5.5 ± 0.7
Smokers (%)	5.7	6.1
Prevalence of type 2 diabetes (%)	5.4	6.3

BMI, body mass index. *$p < 0.05$, vs A allele. Mean ± SD.

Supplementary Table 3. Association of m.1382C with T2D among MEC Japanese Americans-haploid analysis.

	N (Cases/Controls)	m.1382C Freq	Beta *	OR*	95% CI*	*p* value*
Males	1,810 (1,016/794)	0.076	0.3894	1.48	1.01-2.15	0.0424
Females	1,577 (783/794)	0.068	-0.188	0.83	0.53-1.28	0.4014

* The results above are adjusted for age and BMI.

Supplementary Table 4. Factors associated with the risk of type 2 diabetes in male with the m.1382 A allele in J-MICC.

Variables	B	*p* value	OR	(95% CI)
Age (years old)	0.051	< 0.001	**1.05**	(1.039 1.065)
BMI (kg/m^2)	0.069	< 0.001	**1.07**	(1.038 1.105)
Constant	6.726	< 0.001		

Step-up logistic regression analysis. Other input variables were smoking (yes), $p = 0.459$; moderate-to-vigorous intensity physical activity (min/day), $p = 0.595$.

Supplementary Table 5. Factors associated with the risk of type 2 diabetes in male with the m.1382 C allele in J-MICC.

Variables	B	*p* value	OR	(95% CI)
Age (years old)	0.091	< 0.001	**1.10**	(1.048 1.144)
MVPA (min/day)	0.023	0.035	**0.98**	(0.956 0.998)
Constant	6.738	< 0.001		

Step-up logistic regression analysis. MVPA, moderate to vigorous intensity physical activity. Other input variables are body mass index (kg/m2), p = 0.128; smoking (yes), p = 0.195.

Supplementary Table 6. Characteristics of J-MICC subjects by m.1382A>C polymorphism divided with physical activity.

MVPA tirtile	T1			T2			T3		
**m.1382 genotype**	**A**	**C**	***p***	**A**	**C**	***p***	**A**	**C**	***p***
Male									
(n)	1,529	124		1,536	119		1,538	117	
Age (years old)	58.4 ± 0.2	57.8 ± 0.8	0.443	55.3 ± 0.2	53.9 ± 0.7	0.070	56.2 ± 0.2	55.6 ± 0.8	0.498
Height (cm)	167.2 ± 0.2	167.1 ± 0.6	0.909	167.4 ± 0.2	167.4 ± 0.5	0.955	166.2 ± 0.1	167.4 ± 0.5	0.034
BMI (kg/m^2)	24.3 ± 0.1	24.5 ± 0.3	0.605	24.2 ± 0.1	24.3 ± 0.3	0.726	24.1 ± 0.1	23.9 ± 0.3	0.461
Waist circumference (cm)	87.4 ± 0.2	87.2 ± 0.9	0.833	86.1 ± 0.2	85.7 ± 0.8	0.554	85.2 ± 0.2	85.0 ± 0.7	0.750
HbA1c (%)	5.3 ± 0.8	5.4 ± 0.8	0.222	5.3 ± 0.8	5.3 ± 0.7	0.951	5.3 ± 0.8	5.3 ± 1.1	0.456
Smokers (%)	43.1%	43.5%	0.497	35.3%	41.2%	0.117	28.7%	30.8%	0.349
Prevalence of type 2 diabetes (%)	11.2%	18.5%	0.014	10.0%	12.6%	0.218	11.4%	7.7%	0.141
Female									
(n)	2,105	190		2,115	183		2,136	161	
Age (years old)	57.4 ± 0.2	57.2 ± 0.6	0.823	54.4 ± 0.2	55.2 ± 0.6	0.235	55.0 ± 0.2	55.0 ± 0.6	0.995
Height (cm)	154.6 ± 0.1	154.0 ± 0.4	0.153	155.0 ± 0.1	155.1 ± 0.4	0.774	154.1 ± 0.1	154.3 ± 0.4	0.589
BMI (kg/m^2)	23.1 ± 0.1	23.5 ± 0.3	0.102	22.5 ± 0.1	22.4 ± 0.2	0.617	22.6 ± 0.1	22.7 ± 0.2	0.978
Waist circumference (cm)	82.9 ± 0.2	83.5 ± 0.8	0.388	80.6 ± 0.2	80.2 ± 0.7	0.559	80.2 ± 0.2	80.3 ± 0.7	0.910
HbA1c (%)	5.2 ± 0.7	5.2 ± 0.5	0.143	5.2 ± 0.6	5.2 ± 0.6	0.441	5.2 ± 0.6	5.2 ± 0.7	0.781
Smokers (%)	9.8%	13.2%	0.091	8.2%	9.3%	0.349	6.9%	5.0%	0.225
Prevalence of type 2 diabetes (%)	5.1%	3.7%	0.258	3.7%	4.4%	0.377	5.3%	3.1%	0.151

MVPA, moderate vigorous physical activity. BMI, body mass index. Mean ± SD.

Supplementary Table 7. Characteristics of J-MICC subjects by the m.4883C>T polymorphism (haplogroup D).

	C	T
Male		
n	3,108	1855
Age (years old)	56.6 ± 8.2	56.4 ± 8.1
Height (cm)	166.9 ± 6.0	167.1 ± 6.0
BMI (kg/m^2)	24.2 ± 2.9	24.2 ± 3.1
Waist circumference (cm)	86.2 ± 7.9	86.2 ± 8.2
HbA_{1c} (%)	5.3 ± 0.8	5.3 ± 0.9
Smokers (%)	35.7	36.2
Prevalence of type 2 diabetes (%)	10.8	11.4
Female		
n	4,262	2628
Age (years old)	55.6 ± 8.2	55.6 ± 8.2
Height (cm)	154.6 ± 5.4	154.4 ± 5.4
BMI (kg/m^2)	22.8 ± 3.2	22.8 ± 3.2
Waist circumference (cm)	81.3 ± 9.4	81.2 ± 9.6
HbA_{1c} (%)	5.2 ± 0.6	5.2 ± 0.6
Smokers (%)	7.8	9.3*
Prevalence of type 2 diabetes (%)	4.8	4.4

BMI, body mass index. *$p < 0.05$. Mean ± SD.

Supplementary Table 8. Characteristics of J-MICC subjects by the m.5178C>A polymorphism (haplogroup D).

	**C**	**A**
Male		
n	3,112	1851
Age (years old)	56.6 ± 8.2	56.4 ± 8.1
Height (cm)	166.9 ± 6.0	167.1 ± 6.0
BMI (kg/m^2)	24.2 ± 3.0	24.2 ± 3.0
Waist circumference (cm)	86.2 ± 7.9	86.2 ± 8.2
HbA$_{1c}$ (%)	5.3 ± 0.8	5.3 ± 0.9
Smokers (%)	35.7	36.3
Prevalence of type 2 diabetes (%)	10.8	11.3
Female		
n	4,265	2625
Age (years old)	55.6 ± 8.2	55.6 ± 8.2
Height (cm)	154.6 ± 5.4	154.4 ± 5.4
BMI (kg/m^2)	22.8 ± 3.2	22.8 ± 3.2
Waist circumference (cm)	81.3 ± 9.4	81.2 ± 9.6
HbA$_{1c}$ (%)	5.2 ± 0.6	5.2 ± 0.6
Smokers (%)	7.8	9.3*
Prevalence of type 2 diabetes (%)	4.8	4.4

BMI, body mass index. *$p < 0.05$. Mean ± SD.

Supplementary Table 9. Characteristics of J-MICC subjects by the m.3010C>A polymorphism (haplogroup D4).

	G	A
Male		
n	3,287	1676
Age (years old)	56.6 ± 8.2	56.5 ± 8.1
Height (cm)	166.9 ± 6.1	167.1 ± 5.9
BMI (kg/m^2)	24.2 ± 2.9	24.2 ± 3.1
Waist circumference (cm)	86.2 ± 7.9	86.3 ± 8.2
HbA_{1c} (%)	5.3 ± 0.8	5.3 ± 0.9
Smokers (%)	35.8	36.0
Prevalence of type 2 diabetes (%)	10.9	11.2
Female		
n	4,504	2386
Age (years old)	55.6 ± 8.2	55.6 ± 8.3
Height (cm)	154.6 ± 5.4	154.4 ± 5.4
BMI (kg/m^2)	22.8 ± 3.2	22.8 ± 3.2
Waist circumference (cm)	81.2 ± 9.4	81.2 ± 9.6
HbA_{1c} (%)	5.2 ± 0.6	5.2 ± 0.6
Smokers (%)	7.8	9.5*
Prevalence of type 2 diabetes (%)	4.8	4.3

BMI, body mass index. *$p < 0.05$. Mean ± SD.

Supplementary Table 10. Characteristics of J-MICC subjects by the haplogroup D4b (m.1382C or m.15440C).

	Others	D4b
Male		
n	4,514	449
Age (years old)	56.6 ± 8.2	56 ± 8.5
Height (cm)	166.9 ± 6.0	167.2 ± 5.7
BMI (kg/m2)	24.2 ± 3.0	24.3 ± 3.2
Waist circumference (cm)	86.2 ± 7.9	86.1 ± 8.6
HbA1c (%)	5.3 ± 0.8	5.3 ± 0.9
Moderate to vigorous physical activity (min/day)	21.7 ± 17.5	21.7 ± 20.4
Smokers (%)	35.7	37.6
Prevalence of type 2 diabetes (%)	10.9	12.5
Female		
n	6,238	652
Age (years old)	55.6 ± 8.2	55.5 ± 8.2
Height (cm)	154.5 ± 5.4	154.5 ± 5.6
BMI (kg/m2)	22.8 ± 3.2	22.9 ± 3.4
Waist circumference (cm)	81.2 ± 9.4	81.1 ± 9.8
HbA1c (%)	5.2 ± 0.6	5.2 ± 0.6
Moderate to vigorous physical activity (min/day)	18.8 ± 13.5	17.8 ± 12.7
Smokers (%)	8.2	9.8
Prevalence of type 2 diabetes (%)	4.7	3.5

BMI, body mass index. Mean ± SD.

Supplementary Table 11. Factors associated with the risk of type 2 diabetes in the J-MICC subjects divided with MVPA.

MVPA tietile		T1			T2			T3	
Male									
	Age	1.04	(1.019-1.062)	Age	1.082	(1.058-1.106)	Age	1.05	(1.029-1.071)
	BMI	1.07	(1.021-1.122)	BMI	1.07	(1.014-1.130)	BMI	1.074	(1.016-1.134)
	m.1382A>C	1.837	(1.131-2.983)	Constant	0		Constant	0.001	
	Constant	0.002							
Female									
	Age	1.068	(1.038-1.099)	Age	1.089	(1.056-1.123)	Age	1.088	(1.059-1.118)
	BMI	1.168	(1.120-1.219)	BMI	1.217	(1.144-1.295)	BMI	1.083	(1.018-1.151)
	Constant	0		Constant	0		Constant	0	

MVPA, moderate-vigorous physical activity. Values are odds ratio (95%CI). SNPs [m.4883C>T (haplogroup D), m.5178C>A (haplogroup D), m.3010G>A (haplogroup D4), m.15440T>C (haplogroup D4b1), and m.1382A>C (haplogroup D4b2)].
All logistic regression models included the following covariates: age, BMI, smoking (yes), and D4b haplogroup.
